# The challenging diagnosis and treatment of duodenal diverticulum perforation: a report of two cases

**DOI:** 10.1186/s12876-019-1154-2

**Published:** 2020-01-08

**Authors:** Moysis Moysidis, Daniel Paramythiotis, Anestis Karakatsanis, Evropi Amanatidou, Elisavet Psoma, Xanthippi Mavropoulou, Antonios Michalopoulos

**Affiliations:** 1 1st Propedeutic Surgery Department, AHEPA University Hospital of Thessaloniki, St Kiriakidi 1, 54621 Thessaloniki, Greece; 2 Department of Radiology, AHEPA University Hospital of Thessaloniki, Thessaloniki, Greece

**Keywords:** Duodenal diverticulum, Perforation, Emergency surgery, Acute abdomen, Upper GI, Case report

## Abstract

**Background:**

The duodenum is a common site for diverticulum formation. Most of the duodenal diverticula are asymptomatic, incidental findings. Perforation is a rare but potentially lethal complication of duodenal diverticular disease. Surgery remains the mainstay of treatment for perforated duodenal diverticula. In recent years, a few cases were successfully managed either conservatively or with endoscopy.

**Case presentation:**

We present two cases of female patients treated in our department for duodenal diverticulum perforation. The first case was treated surgically with a diverticulectomy. The second case was managed conservatively with bowel rest and intravenous antibiotics. Both patients had an uncomplicated postoperative course and were discharged home.

**Conclusions:**

Both surgical and conservative treatments are viable options for a perforated duodenal diverticulum in selected patients. Patients with a contained duodenal diverticular perforation can be managed conservatively at the outset. Possibly, the introduction of a classification system for duodenal diverticulum perforation may help clinicians in making essential therapeutic decisions.

## Background

Since the first report of a complicated duodenal diverticulum in 1710 by Chomel [1] a great effort has taken place to deepen our knowledge on the subject. The prevalence of duodenal diverticula is believed to be as high as 22% of the population as found in cadaveric studies, increasing with age [2], but the majority of them remain uncomplicated and are only discovered incidentally during endoscopic or imaging studies of the upper GI. Only a small percentage of 1–5% of Duodenal Diverticula (DD) will cause symptoms such as pain, hemorrhage, inflammation (diverticulitis), jaundice, cholangitis or, in especially rare cases, perforation [3, 4].

Perforation of a DD, although the rarest, is the gravest complication, as it bears a high mortality rate, varying from 3 to 30% in the literature [4, 5]. Due to its rarity, it is often overlooked in the differential diagnosis. In fact, it is misdiagnosed in a lot of cases, and its symptoms are attributed to other causes of acute abdomen. Treatment of the perforated DD traditionally used to be surgical. First described in 1963 by Shackleton, the non-operative treatment has become a viable option in recent years and selected patients [6]. More recently, there are some patients who were successfully treated with endoscopy alone, or in combination with surgery [3]. Herein, we report two cases of patients with perforated duodenal diverticula who were treated in our department, one surgically and the other one conservatively, and discuss the rationale behind different options and the evidence available in the literature to assist our clinical decision.

## Case presentation

### Case 1

A 51-years old female patient was admitted through the emergency department with abdominal pain of acute onset, mainly epigastric with a right lumbar reflection. The patient had already visited a private medical facility, where she underwent a CT-scan of the abdomen with oral and IV contrast. The findings were consistent with a duodenal perforation and the clinicians referred the patient to the emergency department for further diagnosis and treatment. The patient did not complain of fever, vomit or nausea. Also, she denied any history of NSAIDs and steroid use or a history of ulcer disease, nor did she describe related symptoms.

**Fig. 1:**
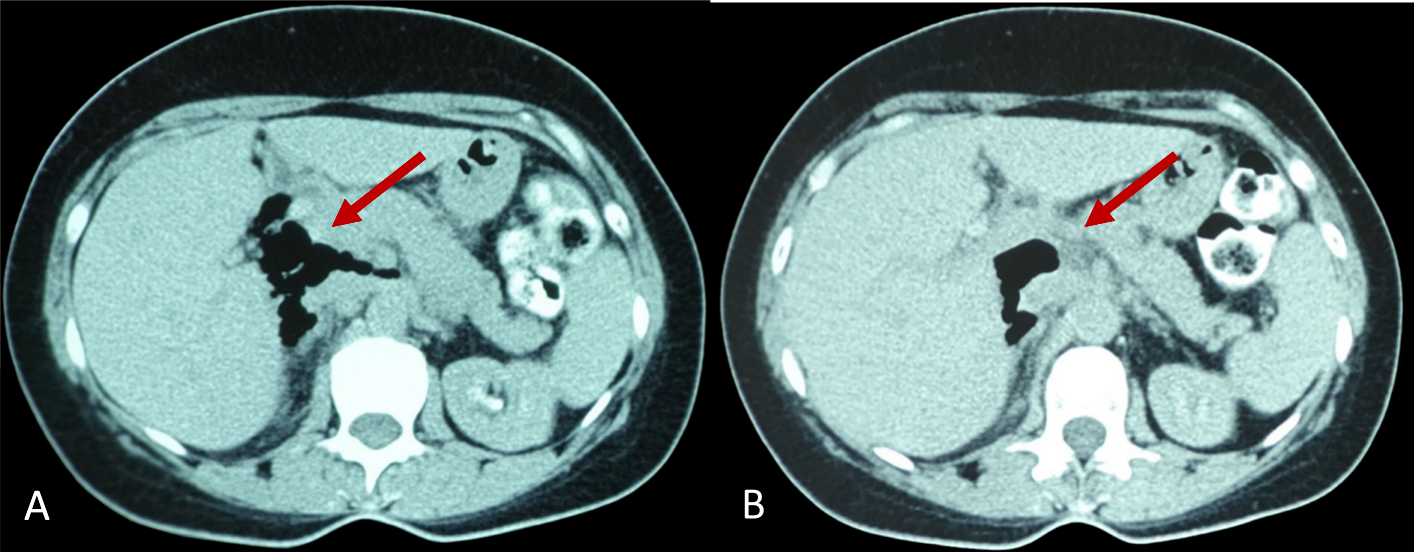
**a**-**b**: CT scan of the abdomen, showing free locules of air in the hepatic hilum and the lesser omental sac

On clinical examination, the patient had a soft abdomen, mild epigastric tenderness with no signs of peritoneal irritation. The patient’s vital signs were normal. She mentioned a medical history of Hashimoto disease under treatment. We proceeded on laboratory testing with the following findings: WBC: 12.93 K/μL, Neut: 76.1%, Hb: 131 g/L, Hct: 36.1%, C-reactive protein on a level of 212.8 mg/l and d-dimmers: 855 ng/ml. Consequently, a new CT scan of the abdomen revealed free air located in the hepatic hilum, the retroperitoneal follicle and the upper liver surface (Fig. 1a, b).

**Fig. 2:**
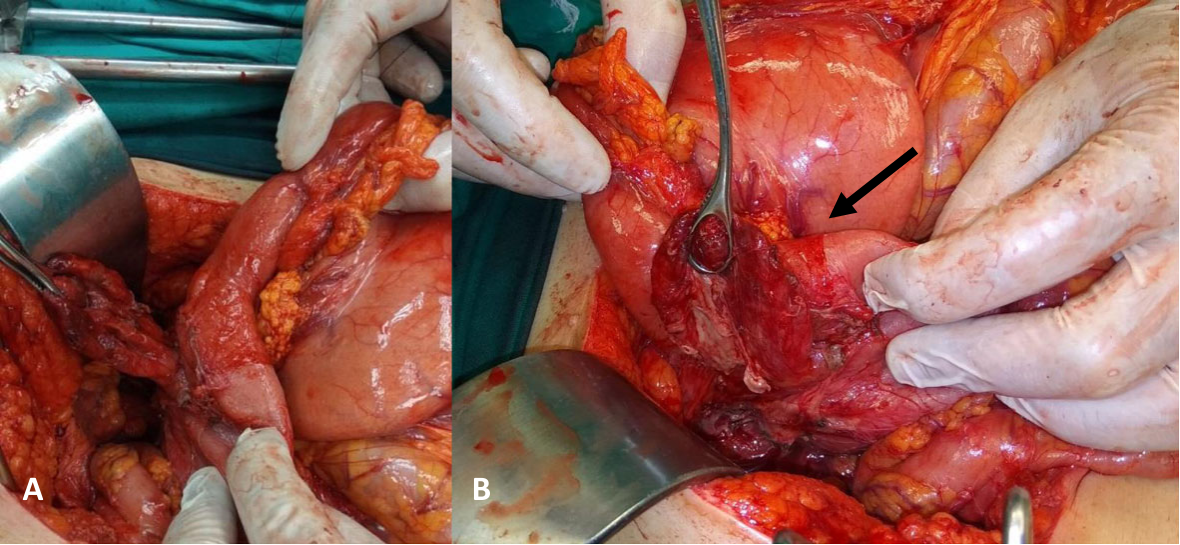
Intra-operative view of the perforated duodenal diverticulum, **a**) Anterior view, **b**) Posterior view, where the site of the perforation was found (arrow), and the diverticulum was covered with pseudomembranes

We decided to perform an immediate exploratory laparotomy during which we noted the presence of a perforated duodenal diverticulum on the second part of the duodenum (Fig. 2a-b). A diverticulectomy was performed with the use of a linear stapler along with the placement of a drain tube in the anatomical area of the second part of the duodenum. The diverticulum was sent for pathology. A Naso-Gastric tube was used and the patient returned to the ward. She stayed at nil per os until hospital day (HD) 8 and started treatment with intravenous antibiotics and PPIs. On post-operative laboratory tests, we noted an immediate drop of WBC (8.37 K/μL). The highest drainage measurement per day was 200 ml on hospital day 2 and the NG tube measurement ranged from 100 to 600 ml per day.

The pathology report confirmed the presence of a small intestine diverticulum with a partial perforation of its wall, with no signs of malignancy. On Hospital Day 8 the patient underwent a radiological small intestine transit with gastrographine, as a part of our department’s protocol for upper GI perforation, before initiating oral feed. In this study, the absence of a duodenal diverticulum in the second part of the duodenum was proved and no signs of leakage could be identified. After that, the NG tube was removed and the patient gradually started oral feeding. The next day the drainage was removed too.

The patient was discharged on hospital day 10, stable with no symptoms of pain with advice for alimentation and post-operative reassessment.

### Case 2

A 58-year old female patient was referred to the emergency department from a District General Hospital with the diagnosis of a perforated duodenal diverticulum in the second part of the duodenum. The patient was admitted through the emergency department of the above hospital with sudden epigastric pain and was hospitalized for 5 days. She underwent an abdominal CT-scan that showed a lesion in the anatomical area of the pancreatic head with air locules and inflammation, findings that were non-specific for a certain clinical entity. In order to discern the exact pathology, an MRI scan was then requested, which showed a diverticulum close to the ampulla of Vater. She was referred for further treatment. The patient’s medical history included Hypertension, Hypothyroidism and Hyperlipidemia, all under treatment. On clinical examination, she had epigastric tenderness and no signs of peritoneal irritation.

**Fig. 3:**
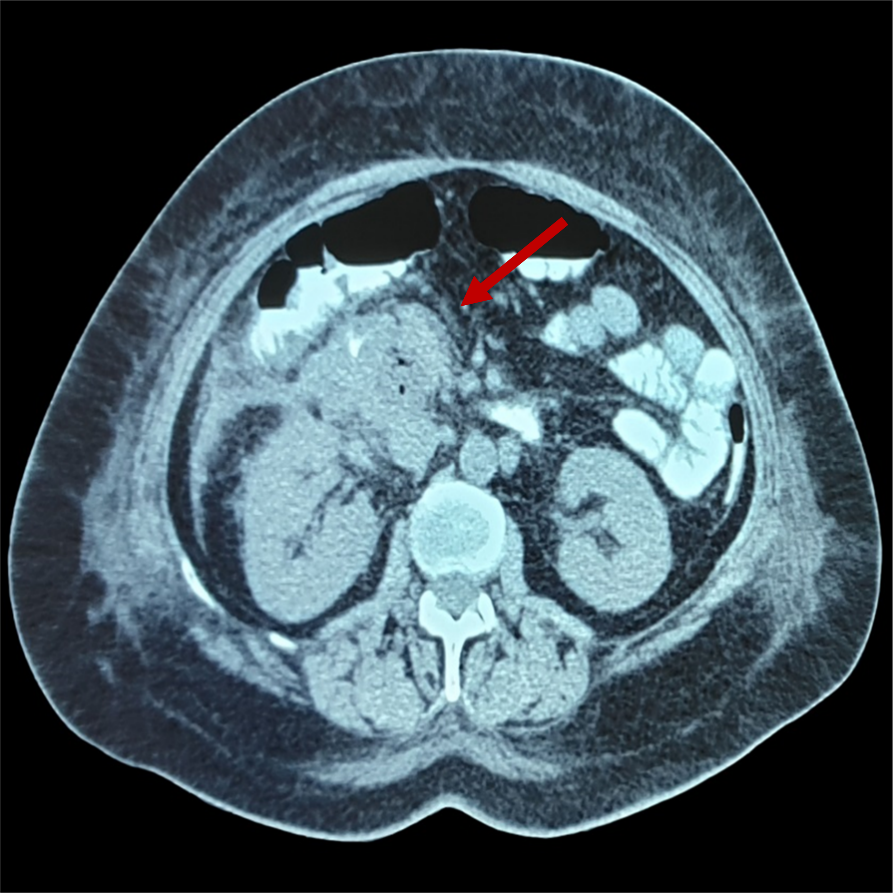
A CT scan of the abdomen shows a 5 cm abscess by the head of the pancreas

On admission, her vital signs were normal, with no fever and her laboratory tests showed: Potassium: 3.4 mmol/l, WBC: 14.33 K/μL with 77.9% neut and 11.1% lymph, Hb: 10.3 g/dL and Hct: 31.3%. We decided to order a new CT scan that revealed free fluid at the sub-hepatic space, spleen and the right paracolic gutter and an abscess of 5 cm diameter near the head of the pancreas (Fig. 3). We decided to proceed with a conservative approach and she was immediately set under treatment which consisted of metronidazole, tigecycline, tinzaparin, paracetamol. The patient stayed at nil per os until hospital day 9 and she received daily 1 L of parenteral nutrition until HD 22.

On patient’s laboratory tests on hospital day 2 we noticed high inflammatory markers (C-reactive protein at 73.10 mg/l and WBC count at 11.36 K/μL) with a Procalcitonin level of 0.2 ng/ml. The WBC count went back to normal on HD 8. On HD 7 the patient was complicated with cough and fever up to 38.4 °C so she was treated for a viral upper respiratory tract infection with oseltamivir 75 mg 12hourly along with ipratropium-salbutamol as required, until HD 12. The patient started oral feeding gradually from HD 10 following an upper GI transit with oral contrast, negative for extra-luminal spillage.

**Fig. 4:**
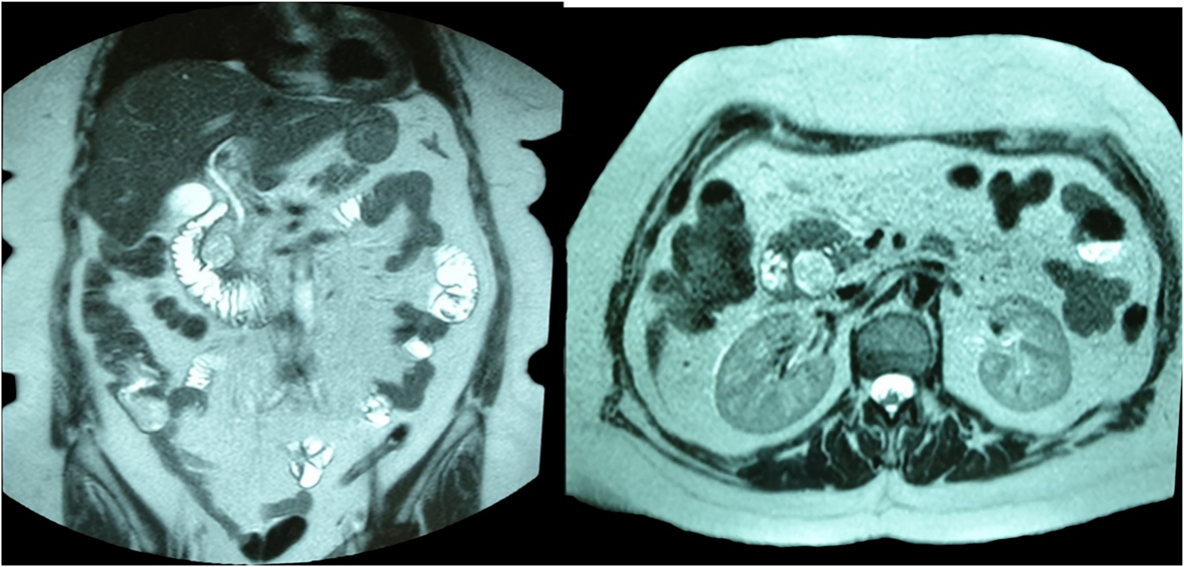
MRI of the Abdomen on HD 19. Images reveal the duodenal diverticulum in the second part of the duodenum with mild inflammation of the surrounding tissues. There is a significant improvement of the imaging findings compared to the initial presentation

Prior to her discharge, on hospital day 18, we repeated the abdominal CT scan on which we noted the presence of the duodenal diverticulum now being clearly shaped with fluid traces on the second part of the duodenum. The above findings are suggestive of significant improvement. On HD 19 the patient underwent an abdominal MRI that also confirmed the presence of a duodenal diverticulum on the second part of the duodenum with a mild inflammation (Fig. 4). The patient continued on conservative treatment with no signs of recurrence. Her hospital stay was complicated by an upper respiratory tract infection by the influenza virus and treated with oseltamivir. She was finally discharged home on hospital day 26 free of symptoms.

## Discussion and conclusions

The duodenum is described as the second most common location for intestinal diverticula only surpassed by the colon [6]. Regarding the DD, they are more frequently situated in the second part, especially on the medial wall, around the ampulla of Vater. Their incidence increases with age and they show no sex predisposition. Most of them appear solitary, roughly 85–90% [4]. In the existing case series, the main causes of DD perforation appear to be diverticulitis (62%), enterolithiasis (10%), iatrogenic (5%), ulceration (5%), trauma (4%) and foreign bodies [5, 6]. Although rare, perforation bears a high mortality rate of 20–34% in older series. Thorson et al. report 8% mortality in a review of 61 cases from 1989 to 2011 and Mathis et al. as low as 3% in a series of 34 patients treated in a single center from 1969 to 2001 [4, 6, 7].

Presenting symptoms from a perforated DD may vary and will not, in most cases be pathognomonic. Pain is the leading symptom that will drive the patient to seek medical help. In the case of intra-peritoneal perforation, it will be abdominal, located at the right upper quadrant or the epigastrium, as in the cases presented here. Some patients may complain of back pain, especially if the perforation is retroperitoneal. Other symptoms will be fever, nausea or vomiting. Some patients will report a long history of vague signs and symptoms which can only be related to the DD retrospectively. Such signs can be weight loss, jaundice, and fullness for a period of months or even years [4–6, 8]. This variety of clinical presentations may puzzle the clinician and thus, high suspicion is required.

Symptoms may easily be attributed to other, more frequent, intra-abdominal pathologies such as cholecystitis, biliary or pancreatic obstruction, pancreatitis, peptic ulcer, retro-cecal appendicitis, neoplasms, pancreatic pseudocyst or even colitis. It is practically almost impossible to differentiate between a perforated duodenal ulcer and perforated DD preoperatively, as the main distinguishing feature will be the fact that duodenal ulcer affects mostly the bulb, while DD will, more often, be located in the second part of the duodenum [6, 9].

On diagnostic workup, laboratory tests will be indicative but not specific for perforation. It seems that in most cases white blood cell count will be elevated with neutrophilia. CRP and PCT levels seem to be useful markers for the diagnosis of perforation and the response to treatment. Their value has been mostly evaluated in cases of sigmoid perforation, but it is suggested that they can be of significance in the follow up of a perforated DD, especially when opting for conservative treatment [3, 5].

**Fig. 5:**
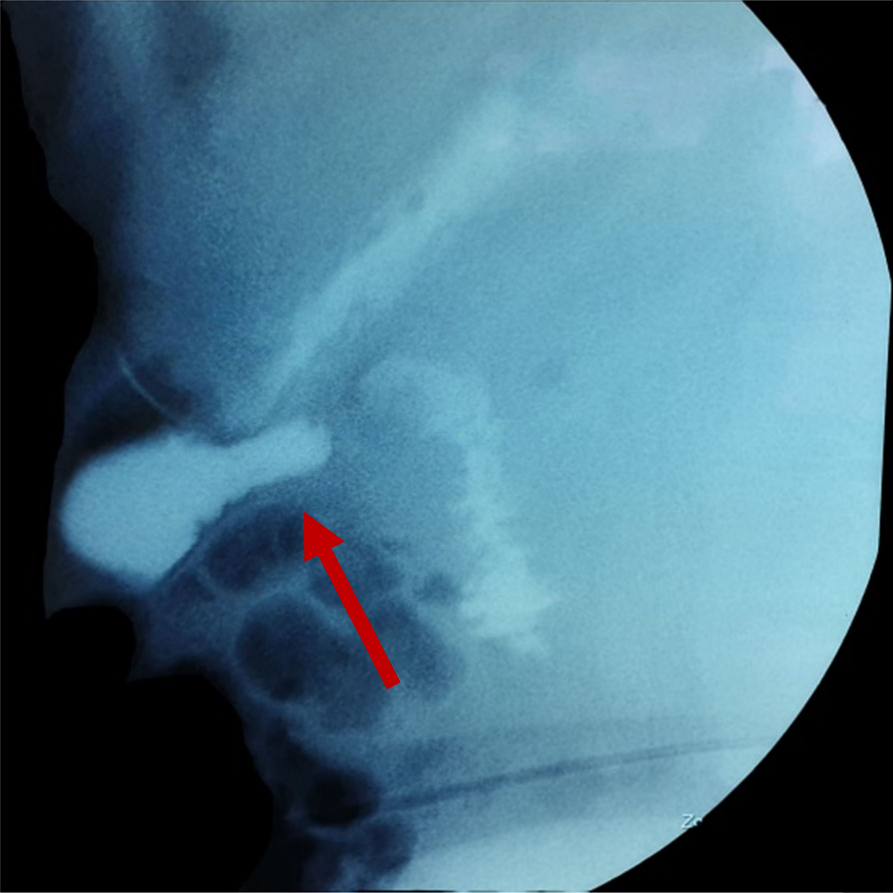
A radiography upper GI series of the second case, demonstrating the windsock sign (arrow)

Clinical imaging is an essential adjunct to our workup of a patient with acute symptoms and, in the majority of cases will make a diagnosis or set the indication for operative treatment. Plain radiography and ultrasound scan have not much to offer in the case of perforated DD, as free sub-diaphragmatic air will appear in about 10% of the cases. One should always keep in mind that retroperitoneal perforation will not cause free intraperitoneal air. Without a doubt, the CT scan of the abdomen is the most useful modality in the diagnosis of a perforated DD [6]. It will be able to identify even small locules of free air in the abdominal cavity, free fluid, fat stranding and the formation of an abscess. All of the above signs may as well be seen in a duodenal ulcer perforation. In a non-emergency setup, an upper GI series is another useful tool in identifying DD, but their ability to demonstrate perforation is low, as it is not sensitive in showing contrast extravasation. The windsock sign in the upper GI series is characteristic of an intra-luminal DD (Fig. 5) [10].

Once the diagnosis of a perforated DD is made, one should choose the ideal treatment option for each patient. Until recently, the only viable option used to be surgery, bearing a considerable mortality rate, as reported previously. A large variety of operations have been described, depending on the gravity of the situation and the location of the diverticulum and the perforation. Diverticulectomy, stapled or hand-sewn, on one or two layers, the use of an omental patch, segmental duodenectomy and duodeno-jejunostomy, duodenal occlusion and biliary diversion, pylorus-preserving Whipple’s procedure are all techniques that have been used in treating a perforated DD [11]. Unfortunately, all the available evidence comes from small case series and case reports, and it is thus difficult to establish a consensus for the surgical treatment. In our case, we opted for a stapled diverticulectomy and a reinforcing layer of sutures, since there was minimal retroperitoneal soiling and the symptoms were initiated only a few hours before.

In keeping with our approach for the second case, there are a number of cases that were treated conservatively with success. The first to ever report such a case was Shackleton in 1963 [12]. Until recently, conservative treatment was reserved for patients with significant co-morbidities and of high perioperative risk. In more recent years, a number of patients with contained perforations with small abscess formation or a few locules of free air were treated with bowel rest, nasogastric tube, antibiotics, intravenous fluids, and total parenteral nutrition, with various levels of success. Some eventually needed surgery, others percutaneous drainage of the abscess cavity [5, 6]. In the case presented here, the good overall condition of the patient, in combination with the small size of the abscess, were the key factors that lead us to the decision to try managing the perforation conservatively.

Advancements in endoscopic techniques and increased experience in endoscopy have offered a third therapeutic option, that of endoscopic intervention. Endoscopic abscess drainage and lavage of the cavity, with or without a drain catheter, has been used alone or before definitive surgical treatment. In the case of the endoscopic approach, the use of CO_2_ gas for inflation is strongly recommended. To the best of our knowledge, only three cases of duodenal diverticulum perforation were treated by endoscopy. This does not constitute enough evidence to safely suggest endoscopic treatment as a sustainable option, as more research is needed to prove the efficacy of the method in the hands of less experienced endoscopists [13].

In recapitulation, albeit uncommon, it has been well established that DD perforation is a serious, potentially lethal complication. In terms of diagnosis and planning, the most valuable modality for the majority of cases is a CT scan with oral and IV contrast in the emergency setting. Surgery is still considered the mainstay of treatment in patients with signs of peritonitis and a free abdominal duodenal leak. A patient with a contained, retroperitoneal leak, with the formation of a small local abscess, without comorbidities or signs of sepsis is a potential candidate for conservative management. The choice is of treatment needs to be individualized, taking into consideration not only the patient factors as described above but the unit’s capability as well, the surgeons’ experience and availability of interventional radiology.

At the time of our review, no formal classification is currently in use to categorize DD perforation in terms of gravity. Stapfer classification for post ERCP perforation is too focused on post endoscopy iatrogenic damage to be used in this case [14]. It is our impression that a classification of a similar philosophy to Hinchey’s classification for the sigmoid diverticular perforation [15] would be of immense aid to the clinicians. It will individualize treatment for each patient, making the decision from a variety of choices easier. The goal of this classification should be to differentiate between local, self-contained inflammation and generalized peritonitis, as well as peritoneal or retroperitoneal perforation.

## Data Availability

The datasets used and/or analyzed during the current study are available from the corresponding author on reasonable request.

## Abbreviations

CRP: C-reactive protein
CT Scan: Computed Tomography scan
DD: Duodenal diverticulum
ERCP: Endoscopic retrograde cholangiopancreatography
GI: Gastrointestinal
HB: Haemoglobin
HD: Hospital Day
PCT: Procalcitonin
WBC: White blood cell
